# Supplementary material for: Alzheimer's disease-like features in resting state EEG/fMRI of cognitively intact and healthy middle-aged APOE/PICALM risk carriers
Source: J Alzheimers Dis. 2025 Mar 17;104(2):509–24. doi: 10.1177/13872877251317489 (PMC12231819; doi:10.1177/13872877251317489)

**Supplemental Material**

**Alzheimer’s disease-like features in resting state EEG/fMRI of cognitively intact and healthy middle-aged *APOE/PICALM* risk carriers**

**Supplemental Figure 1.** A graphical summary illustrating the interaction between group and sex factors in the EEG power spectrum results: delta relative power (A) and low alpha relative power (B).


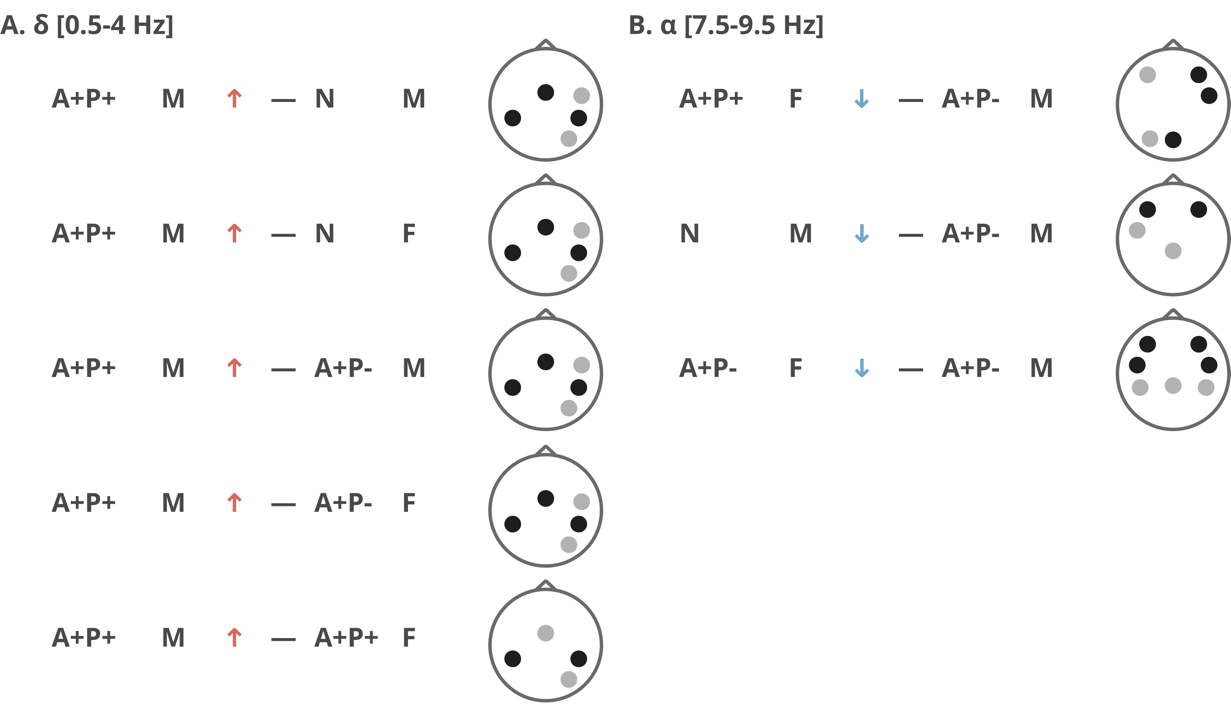


An upward arrow signifies higher power for the first reported group, and a downward arrow signifies lower power. The topographic maps indicate the locations of clusters with significant and near-significant effects. Clusters with significant differences (*p* < 0.05) are marked with black circles, while trend-level differences (*p* < 0.09) are marked with gray circles.

**Supplemental Table 1.** Relative power within the delta band: statistical values and post-hoc information related to Sex*Group interaction differences that are either significant or indicate a trend.

| **Cluster** | **Statistic** | ***p*** | **Post-hoc details** | |
| --- | --- | --- | --- | --- |
| C | 3.53 | <0.05 | M(A+P-) vs. M(A+P+) *p* < 0.01; M(A+P+) vs. F(A+P-) *p* < 0.05; M(A+P+) vs. F(N) *p* < 0.01; M(A+P+) vs. M(N) *p* < 0.05; M(A+P+) vs. F(A+P+) *p* = 0.08 | |
| PTL | 3.25 | <0.05 | F(A+P-) vs. M(A+P+) *p* < 0.01; M(A+P-) vs. M(A+P+) *p* < 0.01; F(A+P+) vs. M(A+P+) *p* < 0.05; M(A+P+) vs. F(N) *p* < 0.01; M(A+P+) vs. M(N) *p* < 0.05 | |
| PTR | 3.64 | <0.05 | F(A+P-) vs. M(A+P+) *p* < 0.01; M(A+P-) vs. M(A+P+) *p* < 0.01; M(A+P+) vs. F(N) *p* < 0.01; M(A+P+) vs. M(N) *p* < 0.01; F(A+P+) vs. M(A+P+) *p* < 0.05 | |
| *Trend level differences* | | | |  |
| CTR | 2.83 | 0.07 | M(A+P-) vs. M(A+P+) *p* < 0.05; M(A+P+) vs. F(N) *p* < 0.01; M(A+P+) vs. M(N) *p* < 0.05; F(A+P-) vs. M(A+P+) *p* < 0.05 | |
| OR | 3.06 | 0.053 | F(A+P-) vs. M(A+P+) *p* < 0.01; M(A+P-) vs. M(A+P+) *p* < 0.01; M(A+P+) vs. F(A+P+) *p* < 0.05; M(A+P+) vs. F(N) *p* < 0.001; M(A+P+) vs. M(N) *p* = 0.06 | |

M: males; F: females; C: central; CTR: central-right; PTL: posterior-left; PTR: posterior-right; OR: occipital-right.

**Supplemental Table 2.** Relative power within the theta and higher alpha band and HFD: statistical values and post-hoc information related to the Sex factor.

| **Cluster** | **Theta**  **(Statistic)** | **Theta**  **(*p*)** | **Alpha-2 (Statistic)** | **Alpha-2**  **(*p*)** | **HFD**  **(Statistic)** | **HFD**  **(*p*)** |
| --- | --- | --- | --- | --- | --- | --- |
| MF | 4.69 | <0.05 | — | — | — | — |
| FL | 5.42 | <0.05 | — | — | 0.16 | 0.069 |
| FR | 4.59 | <0.05 | — | — | 2.23 | 0.14 |
| C | 6.54 | <0.05 | 4.70 | 0.06 | 7.14 | <0.01 |
| CTL | 4.85 | <0.05 | 6.06 | <0.05 | 1.40 | 0.24 |
| CTR | 7.05 | <0.05 | 5.46 | < 0.05 | 5.70 | <0.05 |
| PC | 3.84 | 0.054 | 2.01 | 0.16 | 4.59 | <0.05 |
| PTL | 6.97 | <0.05 | 6.06 | <0.05 | 8.44 | <0.01 |
| PTR | 6.53 | <0.05 | — | — | — | — |
| OC | 6.47 | <0.05 | 7.45 | <0.01 | 6.39 | <0.05 |
| OL | 13.06 | <0.001 | 10.60 | <0.01 | 12.14 | <0.001 |
| OR | 10.30 | <0.01 | 9.76 | <0.01 | — | — |

MF: midfrontal; FL: frontal-left; FR: frontal-right; C: central; CTL: central-left; CTR: central-right; PC: posterior-central; PTL: posterior-left; PTR: posterior-right; OC: occipital-central; OL: occipital-left; OR: occipital-right.

**Supplemental Table 3.** Relative power within the lower alpha band: statistical values and post-hoc information related to Sex*Group interaction differences that are either significant or indicate a trend.

| **Cluster** | **Statistic** | ***p*** | **Post-hoc details** |
| --- | --- | --- | --- |
| FL | 5.29 | <0.01 | F(A+P-) vs. M(A+P-) *p* < 0.05;  M(A+P-) vs. M(N) *p* < 0.05;  M(A+P-) vs. F(A+P+) *p* = 0.09 |
| FR | 5.20 | <0.01 | F(A+P-) vs. M(A+P-) *p* < 0.05;  M(A+P-) vs. F(A+P+) *p* < 0.05;  M(A+P-) vs. M(N) *p* < 0.05 |
| CTL | 5.14 | <0.01 | F(A+P-) vs. M(A+P-) *p* < 0.05;  M(A+P-) vs. M(N) *p* = 0.08 |
| CTR | 4.59 | <0.05 | F(A+P-) vs. M(A+P-) *p* < 0.05;  M(A+P-) vs. F(A+P+) *p* < 0.05 |
| PC | 5.16 | <0.01 | F(A+P-) vs. M(A+P-) *p* = 0.07;  M(A+P-) vs. M(N) *p* = 0.06 |
| PTL | 4.97 | <0.05 | F(A+P-) vs. M(A+P-) *p* = 0.06 |
| PTR | 4.30 | <0.05 | F(A+P-) vs. M(A+P-) *p* = 0.07 |
| OC | 4.51 | <0.05 | M(A+P-) vs. F(A+P+) *p* < 0.05 |
| OL | 4.29 | <0.05 | M(A+P-) vs. F(A+P+) *p* = 0.07 |
| OR | 4.97 | <0.05 | — |

M: males; F: females; FL: frontal-left; FR: frontal-right; CTL: central-left; CTR: central-right; PC: posterior-central; PTL: posterior-left; PTR: posterior-right; OC: occipital-central; OL: occipital-left; OR: occipital-right.

**Supplemental Table 4.** Statistical values and post-hoc information related to Sex*Group interaction in HFD analysis, highlighting significant or trend-level results.

| **Cluster** | ***p*** | **Statistic** | **Post-hoc details** |
| --- | --- | --- | --- |
| *Trend level differences* | | | |
| FL | 0.053 | 3.06 | M(N) > M(A+P+) *p* < 0.01;  M(A+P+) < M(A+P-) *p* < 0.05;  F(N) > M(A+P+) *p* = 0.07 |

M: males; F: females; FL: frontal-left.

**Supplemental Figure 2.** Dependence of Higuchi’s Fractal Dimension value on the parameter *k*_max_.


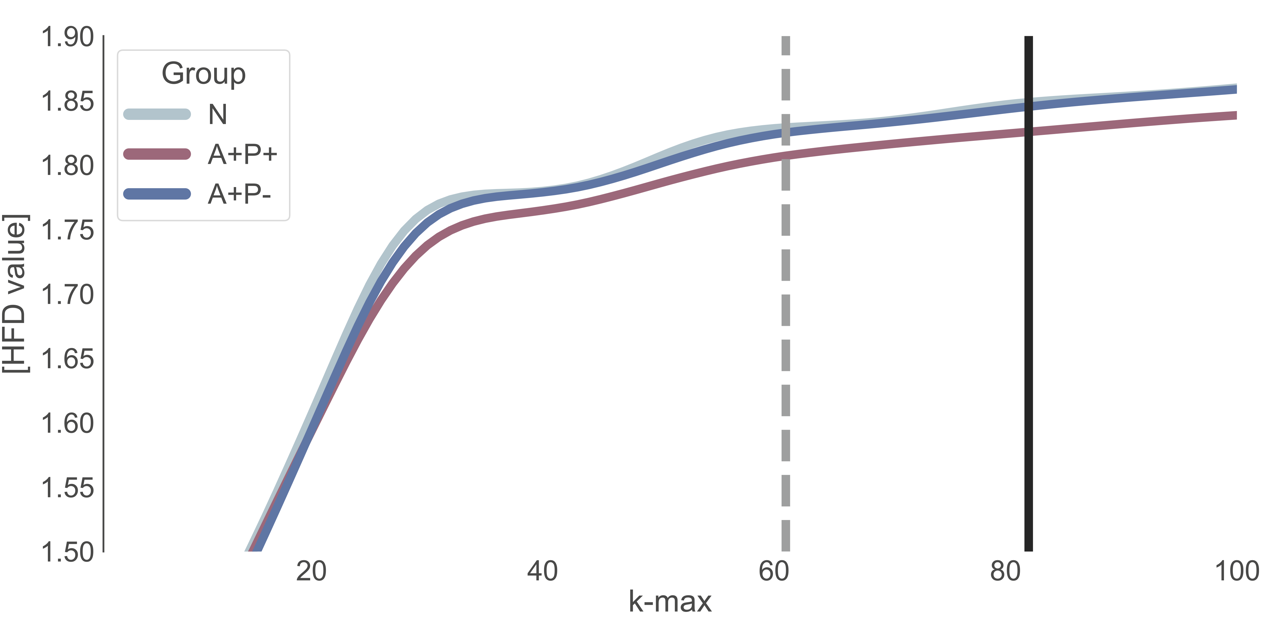


The dotted line indicates the start of HFD stability, and the black line indicates the *k*_max_ selected for the between-group comparison of the HFD measure.

**Supplemental Figure 3.** Differences in connectivity, measured by coherence, were analyzed across the 19 electrodes in the classical 10-20 montage for each frequency band.


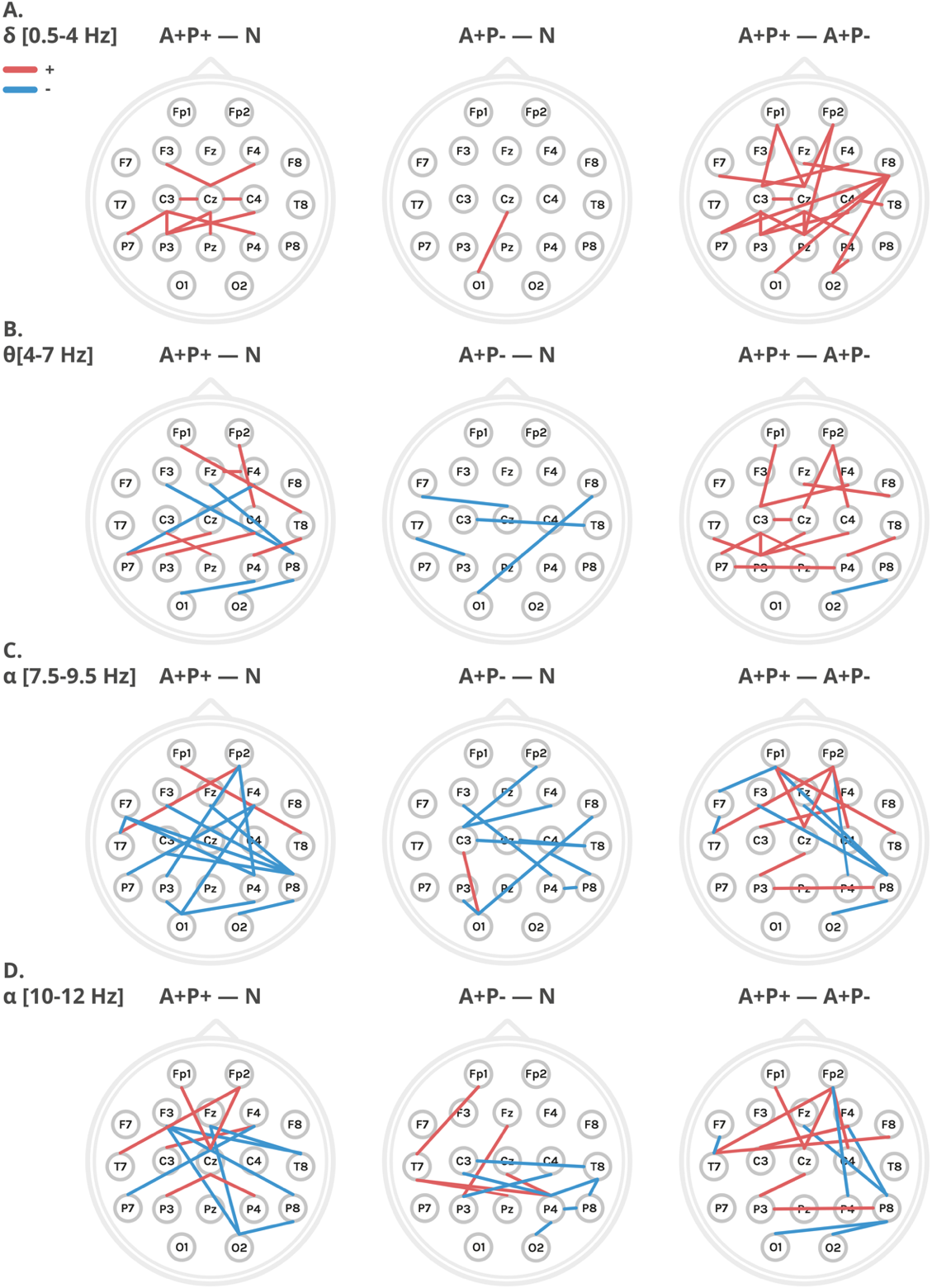


Red lines represent higher coherence for the first group compared to the second group (e.g., A+P+ versus N), while blue lines represent lower coherence. The image displays only significant t-test results (Fieldtip) between the electrodes, but none of these retained significance after FDR correction.

**Supplemental Figure 4.** EEG connectivity (coherence) using a high-density montage with all 128 electrodes.


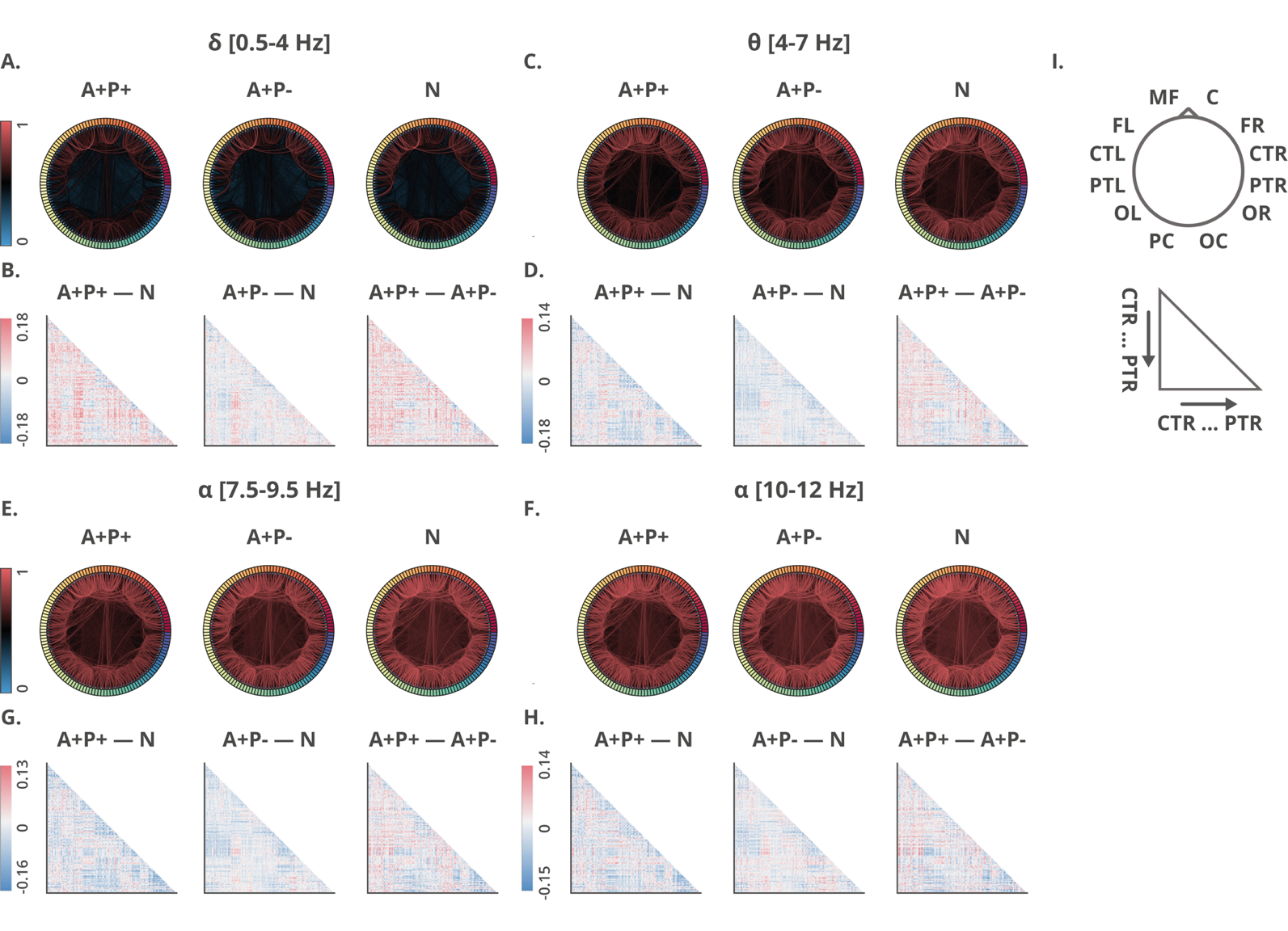


Connectograms for each group are shown, with distinct frequency bands in panels labeled as A, E, C, and F in the graphs. Matrix representations highlighting group differences are displayed in graphs B, D, G, and H. Graph I serves as a legend, detailing electrode placement in all plots, arranged according to previously defined clusters.

**Supplemental Table 5.** Mapping of the 21 Independent Components to neural networks.

| **Neural network** | **Independent component No and its correlation coefficient with the given network** |
| --- | --- |
| Default Mode Network | **11 (r=0.38)**, 5 (r=0.34), 20 (r=0.11) |
| Sensorimotor Network | **21 (r=0.46)**, 16 (r=0.43), 19 (r=0.24) |
| Visual Network | **12 (r=0.56)**, 15 (r=0.41), 13 (r=0.25) |
| Salience Network | **2 (r=0.35)**, 10 (r=0.10), 14 (r=0.08) |
| Dorsal Attention Network | **19 (r=0.38)**, 10 (r=0.27), 13 (r=0.10) |
| Fronto-Parietal Network | **6 (r=0.20)**, 17 (r=0.17), 4 (r=0.14) |
| Language Network | **3 (r=0.39)**, 8 (r=0.21), 10 (r=0.11) |
| Cerebellar Network | **1 (r=0.37)**, 18 (r=0.34), 17 (r=0.20) |

**Supplemental Figure 5.** The representation of IC 5, IC 11, and IC 20 – components with the highest correlation coefficients with the Default Mode Network (DMN). Retrieved from the CONN toolbox.


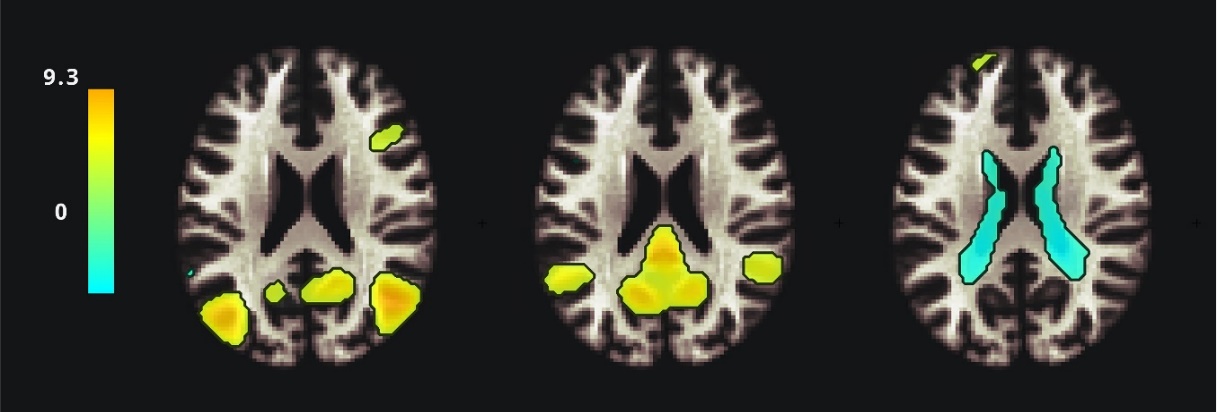

Supplement: sj-docx-1-alz-10.1177_13872877251317489 - Supplemental material for Alzheimer's disease-like features in resting state EEG/fMRI of cognitively intact and healthy middle-aged APOE/PICALM risk carriers [file sj-docx-1-alz-10.1177_13872877251317489.docx]
